# Cæsarean Section

**Published:** 1885-10

**Authors:** R. O. Engraham

**Affiliations:** Member of the Section on Gynecology for the Third District, Montezuma, Ga.


### CÆSAREAN SECTION.*

BY R. O. ENGRAHAM, M. D., MEMBER OF THE SECTION ON GYNECOLOGY FOR THE THIRD DISTRICT, MONTEZUMA, GA.

H. T., aged 38, multipara, was taken in labor July 1, 1882, attended by midwife. After unsuccessful efforts to deliver, I was called on the morning of the 4th. I found the right hand of the child outside the external genitals; arm much bruised from traction. I was informed that the hand came down on the first day of labor.

Attempting to introduce my hand, it was met immediately in front of the os by a large, unyielding globose tumor. Was informed that patient's bowels and bladder had acted two hours previously.

She was considerably exhausted and contractions quite feeble. I attempted to push aside the tumor and return the presenting hand and perform version, but from the tumor impinging I was unable to do so. I decided, if possible, to amputate the arm, eviscerate and use Braun's cephalstrite. I called in consultation Dr. A. A. Watts, who assisted. After the patient was under the influence of an anesthetic (chloroform), Dr. Watts made an unsuccessful attempt to return the protruding hand. After the most thorough efforts to perform craniotomy without being successful, we debated the propriety of excision of the tumor, but after thorough examination as could be made with the arm of the child in the vagina, and the pressure of the super-imposed trunk and head, we decided that our patient would have a better chance for recovery from cæsarean section, in her exhausted condition, than from craniotomy and excision of tumor combined.

She was placed recumbent upon mattress, fully chloroformed; instruments, sponges, etc., were immersed in a two per cent. solution of carbolic acid.

*Read by title at the Thirty-sixth Annual Session of the Medical Association of Georgia April 15, 1885, at Savannah.

An incision was made one inch below the umbilicus with a pointed bistoury. Index finger of left hand was introduced and guarded the point of the knife.

The incision was continued along the linea alba to within one and a half inches of the pubis, making an opening five and a half inches in length. The peritoneum was then lifted upon a tenaculum; an incision made large enough to admit index finger of left hand, which guarded point of knife. An incision was made corresponding nearly to one made in abdominal parietes.

At this juncture, an elastic cord was slipped around the cervix with uterine sound and single, lightly tied knot was made, which was tightened just at the extraction of child.

Reaching the uterus an incision five inches long was made along the median line of the anterior walls. The incision was made so as to avoid the integrity of both the cervix and fundus. The child was found with head in left hypogastrium, face looking towards sacrum and right arm in vagina. Placed my right hand under the right arm-pit by reaching over its back and grasped the left arm with my left hand and lifted it through the opening just made, delivering by vertex. The child was dead. It weighed eight and a half pounds. The loss of blood amounted to a little more than an ordinary labor.

Placing my hand in the uterine cavity, I explored for the offending tumor; found it in posterior walls of uterus, extending two or two and a half inches towards the fundus, and running down towards external os and jutting down against posterior walls of vagina, an inch below the os and about six and a half inches in circumference, evidently uterine fibroid. At this point of the operation the bedstead gave way, precipitating operator, assistant and all to the floor. Dr. Watts, who was administering the anesthetic, notified me that patient's pulse was growing weak. I turned out clots from uterus, sponged out uterine cavity with weak carbolized water, and no blood having escaped into abdominal cavity, I closed uterine incision with three deep and three superficial carbolized silk sutures. Three of the ligatures were placed one inch apart, passing through the uterine walls. The other three were placed, one alternately between the first and passed externally only half through uterine walls. The peritoneum was closed with four sutures. The abdominal incision was closed with eight carbolized silk sutures, and she was nearly half encircled with broad strips of adhesive plaster. She was cleansed, redressed and placed upon another bed. A dressing was applied upon soft linen of carbolized sweet oil, 32 to 8℥.

In an hour patient fully rallied from anesthetic. Morphia was administered, and in three hours left her resting comfortable.

Operation lasted forty-five minutes.

August 5.—Found patient had spent quiet night; pulse 110, temperature 99, with some abdominal fullness. There being some thirst and slight nausea, ordered water acidulated with citric acid, which gave temporary relief. Left morphia to meet any manifestation of pain. Incision seemed to be doing well. Prescribed quinine in anticipation of surgical fever.

August 6.—Considerable distention about abdomen with tympanitis; tongue coated dirty white, with some pain in left hypogastrium; pulse 115, temperature 99 1-2°F. No motion from bowels. Ordered castor oil and turpentine. Ordered carbolized dressing continued. Gave quinia gr. x, carb. ferri gr. 1. Doveri gr. v., every six hours.

August 7.—Suffering considerable; abdomen greatly distended; vomiting frequently, ejecting a dirty looking fluid, filled with particles of sand; tongue dirty brown and dry; pulse 120, temperature 100°F. Bowels have not responded to oil; gave enema; in ten minutes enema returns, bringing away teaspoonful of sand and chalky white fluid. In thirty minutes repeated enema, but it returned as given. After many futile attempts with enemata, I oiled my finger and introduced it well up into the rectum. About three inches within the sphincter I was met by a mass pressing against the anterior wall of the rectum, which evidently was the uterine tumor previously encountered. By gentle rotary motion I passed the tumor, and half inch further my finger was met by what seemed to be impacted fæces of the consistency of partially dried glacier's putty, but it was beyond my reach. After various attempts to dislodge it, I introduced my hand (patient being chloroformed) into the rectum.

After introducing my hand cautiously, I was able to raise the uterine tumor, slip my fingers forward, and with much difficulty break up the mass. With my hand still in the rectum, with a Davidson syringe I directed a strong stream of tepid water against the mass. Withdrawing my hand, in a few minutes quite a quantity of the impacted mass came away, consisting in the main of chalky marl and sand, which patient told me she had ate before she was confined, as she remarked that she had an uncontrollable appetite for it.

Of this marl and sand there were gathered from her stools in all two and a half pounds. After she rallied from the anesthetic, administered hypodermic of morphia; prescribed beef, wine and iron, ordered quinia, gr. x, pulv. Doveri, gr. v, every six hours and continued carbolized oil dressings.

August 8.—Patient comfortable, pulse 100, temperature 99° F.; tongue moist, abdominal distention considerably diminished, cheerful with some desire for food, prescription continued.

August 9.—Patient improving, abdominal distention disappeared, pulse 90, temperature 98 3-5, tongue moist and cleaning off, appetite fair. Ordered treatment continued, except Dovers to be discontinued, and morphia to be given as indication demands.

She continued to improve up to the 13th. Union in the abdominal wound was perfect, except a single suture nearest the umbilical extremity of the incision. At this time I found the pulse 110, temperature 100° F., tongue dry, skin hot and dry. Patient complained of a severe throbbing pain about the re-united part of incision. Parts looked red and swollen. Ordered quinia, gr. xx, Dovers gr. vii, every six hours.

August 14.—Pulse 120, temperature 101, skin hot and dry, restless with great pain of a burning, throbbing character about ununited part of incision. Parts looked red, felt hard as if circumscribed infiltration had occurred one and a half inches external to left lip of incision, opposite ununited point. Prescription continued. Leadwater and laudanum applied to painful point.

August 15.—Somewhat worse, pulse 125, temperature 102, pain controlled only with anodyne. A well defined circumscribed hard lump one and a quarter inches to left of ununited point of incision. Treatment continued.

August 16.—Condition aggravated, pulse 128, temperature 102½. Introduced my finger through opening left in incision, explored to left, found a fluctuating tumor about the size of a billiard ball, which I punctured with a trocar through the abdominal wall from externally over center of tumor, when a discharge of two and a half ounces of unhealthy looking pus escaped. Introduced drainage tube in opening made with trocar. Prescribed quinia, iron and morphia.

August 18.—Much more comfortable, good night's rest, pulse 100, temperature 99½, treatment continued.

August 19.—Improving, pulse 95, temperature 98 3-5, treatment continued.

August 20.—She seems better in every respect, cheerful, says she will be up in a few days, drainage tube removed.

August 21.—Sat up in bed, ate breakfast, one hour later had severe chill, lasting about an hour, fever ensued with pulse 100, temperature 100½, some delirium, prescribed 30 gr. quinine, to be followed with gr. x every six hours, gave brandy cautiously.

August 22.—Mind clear, pulse 95, temperature 99½, quinia and opium.

August 23.—Pulse 110, temperature 101 3-5 with all the indications of sepscis. Prescribed quinia, tincture of iron, opium, brandy. She died on morning of the 24th, with convulsion, on 24th day after the operation.

I was permitted to examine the abdominal cavity enough to find that union had taken place completely in both the peritoneal and uterine incision with sutures in situ. No escape had taken place into abdominal cavity.

*Case 2.*—Although I denominate this case 2d, in reality it is case 1st, as the operation took place several years prior to the one just reported.

This case I will not take the time to go into details, as it was substantially a similar operation, and is reported merely to furnish statistics of the operation.

Mrs. Eugenia R., age 25, primipara, was taken in labor September 18th, 1874. I was called at first signs of approaching labor.

On introducing my finger into vagina, I was quite astonished to find the pelvis fearfully contracted and distorted so badly that it was quite apparent that the head could never pass. Assisted by Drs. Roberts and Stubbs, we performed craniotomy and made every effort to deliver, but our efforts were entirely futile, and we were left no other alternative but hysterotomy, which I proceeded to perform, with their assistance, in the manner just described in previous operation, with no particular accident or variation, except a tendency of intestine to protude from the opening, which was with difficulty controlled. Also, considerable hemorrhage took place and considerable blood entered the abdominal cavity, necessitating sponging out the cavity at the conclusion of the delivery. The badly mutilated fetus was lifted by the feet through the opening from the uterine cavity, and the various incisions sutured, as in previously described operation, with all the antiseptic precautions at my command. Carbolized oil dressing and adhesive strips used as heretofore described.

The case made a good recovery, and she was living the last I knew of her, a few years ago.

